# Comparison of an improved self-consistent lower bound theory with Lehmann’s method for low-lying eigenvalues

**DOI:** 10.1038/s41598-021-02473-y

**Published:** 2021-12-06

**Authors:** Miklos Ronto, Eli Pollak, Rocco Martinazzo

**Affiliations:** 1 Chemical and Biological Physics Department, Weizmann Institute of Science, 76100 Rehovot, Israel; 2 School of Chemistry, University of Leeds, Leeds, LS2 9JT UK; 3 Dipartimento di Chimica, Universitá degli Studi di Milano, 20133 Milan, Italy; 4 Istituto di Scienze e Tecnologie Chimiche “Giulio Natta” (SCITEC), Consiglio Nazionale delle Ricerche, 20133 Milan, Italy

**Keywords:** Quantum chemistry, Chemical physics, Applied mathematics

## Abstract

Ritz eigenvalues only provide upper bounds for the energy levels, while obtaining lower bounds requires at least the calculation of the variances associated with these eigenvalues. The well-known Weinstein and Temple lower bounds based on the eigenvalues and variances converge very slowly and their quality is considerably worse than that of the Ritz upper bounds. Lehmann presented a method that in principle optimizes Temple’s lower bounds with significantly improved results. We have recently formulated a Self-Consistent Lower Bound Theory (SCLBT), which improves upon Temple’s results. In this paper, we further improve the SCLBT and compare its quality with Lehmann’s theory. The Lánczos algorithm for constructing the Hamiltonian matrix simplifies Lehmann’s theory and is essential for the SCLBT method. Using two lattice Hamiltonians, we compared the improved SCLBT (*i*SCLBT) with its previous implementation as well as with Lehmann’s lower bound theory. The novel *i*SCLBT exhibits a significant improvement over the previous version. Both Lehmann’s theory and the SCLBT variants provide significantly better lower bounds than those obtained from Weinstein’s and Temple’s methods. Compared to each other, the Lehmann and *i*SCLBT theories exhibit similar performance in terms of the quality and convergence of the lower bounds. By increasing the number of states included in the calculations, the lower bounds are tighter and their quality becomes comparable with that of the Ritz upper bounds. Both methods are suitable for providing lower bounds for low-lying excited states as well. Compared to Lehmann’s theory, one of the advantages of the *i*SCLBT method is that it does not necessarily require the Weinstein lower bound for its initial input, but Ritz eigenvalue estimates can also be used. Especially owing to this property the *i*SCLBT method sometimes exhibits improved convergence compared to that of Lehmann’s lower bounds

## Introduction

According to the Ritz–MacDonald theorem, the eigenvalues of the Hamiltonian of a quantum mechanical system provide upper bounds for the true energy levels of the system^1,2^. While these eigenvalues typically give sufficiently accurate upper estimates for the energy levels, they provide no information on their quality; that is, a tight bound from below the true energy level. The variational principle for eigenvalues is a fundamental theorem in physics and chemistry and not limited to the energy levels in quantum mechanics only. The first lower bound expression was introduced for vibrating systems by Temple in 1928^3^, which is limited if no experimental data is available. The Weinstein lower bound^4^ is based on the variance associated with the approximate eigenvalues of the Hamiltonian, and Stevenson verified its validity^5^ and further generalized the method^6^. These two approaches are the most important lower bound methods based on the variance of the Hamiltonian and several theoretical studies^7–16^ and practical implementations^17–19^ have improved their performance. A significant step forward was the development of Lehmann’s optimal inclusion intervals, which provided an optimization of the basis set that maximizes Temple’s lower bound^20–23^. Another class of lower bound theories is based on the method of intermediate operators^24,25^. These approaches were first applied to He energy levels using a special choice by Bazley and Fox^26,27^, and motivated several further improvements for Coulombic and other potentials^28–33^. Bracketing functions have also been successfully applied to lower bound problems^34–38^. Further lower bound calculation strategies are also available for He atoms^39–42^. A comprehensive discussion of these methods can be found in Ref.^43^ and a historical review on lower bound theories can be found in Ref.^44^.

Recently, novel lower bound methods^45,46^ have been developed based on the Lánczos construct^47–49^, which significantly improved Temple’s lower bound for energy levels, as tested on quartic oscillators. The Lánczos algorithm provides an orthogonalization of states on the Krylov space, and from the resulting tridiagonal matrix, the approximate eigenvalues and the corresponding variances can be readily determined for the original Hamiltonian. In these novel lower bound methods, the energy factor in Temple’s original lower bound formula is replaced by a “residual energy”, for which, based on the Lánczos construct, a well-defined and rigorous algorithm can be provided. The application of this residual energy enables the calculation of lower bounds for ground and low-lying excited states. As an improvement, Pollak and Martinazzo developed a self-consistent lower bound theory (SCLBT)^50,51^ and applied it for lattice Hamiltonians^50^ and quartic^51^ and double-well^52^ potentials. Using the concept of the residual energy, the SCLBT can systematically improve the quality of lower bounds based on the information from lower bounds for higher-lying eigenvalues: the higher the maximal considered level, the tighter the lower bounds to the levels below. In the present paper, the SCLBT is further improved by defining tighter bounds for certain parameters used in the theory. The performance of the improved SCLBT (*i*SCLBT) is tested and compared with that of the Lehmann lower bounds, using two lattice Hamiltonians.

The remainder of the paper is organized as follows. First, the theory of Weinstein, Temple, SCLBT, and Lehmann lower bound methods is discussed on an equal footing provided by the theoretical framework of the Lánczos construct. Then, the numerical implementation using two lattice Hamiltonians is presented. The lower bounds from the previous implementation of the SCLBT are compared with those from the *i*SCLBT method presented in this paper for the ground-state energy levels of the lattice Hamiltonians. Finally, a comparison of the *i*SCLBT and Lehmann methods is discussed based on lower bounds calculated up to the fourth excited state of the models considered. The paper ends with a summary and the main conclusions.

## Theory

### General considerations

In this section, lower bound theories are considered for the energy levels of quantum mechanical systems and for simplicity, real valued functions are assumed. Let us consider a Hermitian Hamiltonian operator ${\hat{H}}$ with energy eigenstates $|\varphi _{j}\rangle$. The corresponding true energy levels $\varepsilon _j$ can be determined from the time-independent Schrödinger equation

1 $$\begin{aligned} {\hat{H}}|\varphi _{j}\rangle =\varepsilon _j|\varphi _{j}\rangle ,\ \ j=1,2,\ldots , \end{aligned}$$

where the energy eigenvalues are in ascending order, with the ground state denoted by $j= 1$ (instead of $j=0$, typically accepted in the literature). The exact representation of the Hamiltonian operator using an orthonormal basis set $\{|\Psi _{j}\rangle =1,2,\ldots \}$ can be written as

2 $$\begin{aligned} {\hat{H}}=\sum _{\begin{array}{c} j=1 \\ k=1 \end{array}}^{\infty }|\Psi _{j}\rangle {\mathbb {H}}_{jk}\langle \Psi _{k}|, \end{aligned}$$

with

3 $$\begin{aligned} {\mathbb {H}}_{jk}=\langle \Psi _{j}|{\hat{H}}|\Psi _{k}\rangle . \end{aligned}$$

However, in numerical calculations the full Hamiltonian matrix ${\mathbb {H}}$ cannot be used: it needs to be represented in a finite basis set, with *L* states. Let us define a projection operator onto this *L*-dimensional subspace as

4 $$\begin{aligned} {\hat{P}}_{L}=\sum _{j=1}^{L}|\Psi _{j}\rangle \langle \Psi _{j}| \end{aligned}$$

and the projection onto the complementary space as ${\hat{Q}}_{L}$ such that the combination is the identity operator in the full Hilbert space as

5 $$\begin{aligned} {\hat{P}}_{L}+{\hat{Q}}_{L}={\hat{I}}. \end{aligned}$$

The discretized Hamiltonian projected onto the finite basis can be written as

6 $$\begin{aligned} {\hat{H}}_{L}={\hat{P}}_{L}{\hat{H}}{\hat{P}}_{L} \end{aligned}$$

and we assume that it can be diagonalized as

7 $$\begin{aligned} {\hat{H}}_{L}|\Phi _{L,j}\rangle =\lambda _{L,j}|\Phi _{L,j}\rangle ,j=1,\ldots ,L, \end{aligned}$$

where $|\Phi _{L,j}\rangle$ are normalized eigenfunctions and $\lambda _{L,j}$ are real eigenvalues. According to the Ritz–Macdonald variational principle, any $\lambda _{L,j}$ eigenvalue gives an upper bound to the exact eigenvalue $\varepsilon _j$ as

8 $$\begin{aligned} \lambda _{L,j}\ge \varepsilon _j. \end{aligned}$$

For each $\lambda _{L,j}$ eigenvalue, the corresponding variance $\sigma ^2_{L,j}$ is defined as

9 $$\begin{aligned} \sigma ^2_{L,j}=\langle \Phi _{L,j}|\left( {\hat{H}}^{2}-\lambda _{L,j}^{2}{{{\hat{I}}}}\right) |\Phi _{L,j}\rangle . \end{aligned}$$

A central element of the theory is the set of basis vectors $|\Psi _{k}\rangle$ constructed using the Lánczos methodology, which brings the projected Hamiltonian to the form

10 $$\begin{aligned} {\hat{H}}_L=\sum _{k=1}^L\left[ {\mathbb {H}}_{kk}|\Psi _{k}\rangle \langle \Psi _{k}|+{\mathbb {H}}_{k+1,k}|\Psi _{k+1}\rangle \langle \Psi _{k}|+{\mathbb {H}}_{k,k-1}|\Psi _{k}\rangle \langle \Psi _{k-1}|\right] \end{aligned}$$

and the complementary part of the Hamiltonian then has the form

11 $$\begin{aligned} {\hat{Q}}_{L}{\hat{H}}=\sum _{k=L+1}^{\infty }\left[ {\mathbb {H}}_{kk}|\Psi _{k}\rangle \langle \Psi _{k}|+ {\mathbb {H}}_{k+1,k}|\Psi _{k+1}\rangle \langle \Psi _{k}|+ {\mathbb {H}}_{k,k-1}|\Psi _{k}\rangle \langle \Psi _{k-1}|\right] . \end{aligned}$$

Using these expressions, the variance in Eq. (9) can be conveniently rewritten as

12 $$\begin{aligned} \sigma ^2_{L,j}=\langle \Phi _{L,j}|{\hat{H}}{\hat{Q}}_{L}{\hat{H}}|\Phi _{L,j}\rangle =\langle \Phi _{L,j}|\Psi _{L}\rangle ^{2}{\mathbb {H}}_{L,L+1}^{2}, \end{aligned}$$

showing that when using a Lánczos basis set, the variances can be obtained from the matrix elements of the Hamiltonian, without the explicit need to calculate elements of the Hamiltonian squared. Naturally, these are implicit in the Lánczos basis vectors.

### Weinstein and Temple lower bounds

To discuss lower bound theories on an equal formal footing, let us introduce the square of the overlap of the *j*th eigenfunction in the projected space with the exact *k*th eigenfunction as

13 $$\begin{aligned} a_{L,kj}=\langle \varphi _{k}|\Phi _{L,j}\rangle ^2. \end{aligned}$$

Lower bounds can be obtained by using a Cauchy–Schwartz inequality in the form of

14 $$\begin{aligned} |\langle \varphi _{j}|{\hat{Q}}{\hat{H}}|\Phi _{L,j}\rangle | ^{2}\le \langle \varphi _{j}|{\hat{Q}}|\varphi _{j}\rangle \langle \Phi _{L,j}|{\hat{H}}{\hat{Q}}{\hat{H}}|\Phi _{L,j}\rangle , \end{aligned}$$

where ${\hat{Q}}$ is a projection operator. Inserting

15 $$\begin{aligned} {\hat{Q}}={\hat{I}}-|\Phi _{L,j}\rangle \langle \Phi _{L,j}| \end{aligned}$$

into the Cauchy–Schwartz inequality and rearranging gives

16 $$\begin{aligned} \varepsilon _j\ge \lambda _{L,j}-\sigma _{L,j}\sqrt{\frac{1-a_{L,jj}}{a_{L,jj}}}, \end{aligned}$$

and with the assumption that $a_{L,jj}\ge 1/2$, the Weinstein lower bound can be obtained as

17 $$\begin{aligned} \varepsilon _j \ge \varepsilon ^{\mathrm {We}}_{L,j}\equiv \lambda _{L,j}-\sigma _{L,j}. \end{aligned}$$

As discussed in Ref. 51, this assumption is further restricted by the accepted condition for the validity of the Weinstein lower bound^5^:

18 $$\begin{aligned} \lambda _{L,j}\le \frac{\varepsilon _j+\varepsilon _{j+1} }{2}. \end{aligned}$$

To obtain the Temple lower bound formula, let us first define a “residual energy” $\bar{\lambda }_{L,j}$ for each eigenstate in the projected space such that the Ritz eigenvalue can be rewritten as

19 $$\begin{aligned} \lambda _{L,j}=a_{L,jj}\varepsilon _j+\left( 1-a_{L,jj}\right) \bar{\lambda }_{L,j}. \end{aligned}$$

Using the overlap defined in Eq. (13), the residual energy is given by the relation

20 $$\begin{aligned} \bar{\lambda }_{L,j}=\sum _{k=1}^{\infty }\frac{a_{L,kj}\left( 1-\delta _{jk}\right) \varepsilon _{k}}{1-a_{L,jj}}, \end{aligned}$$

where $\varepsilon _{k}$ is the exact eigenvalue and $\delta _{jk}$ is a Kronecker delta. Thus, from Eq. (19) the diagonal elements of the overlap can be written as

21 $$\begin{aligned} a_{L,jj}=\frac{\bar{\lambda }_{L,j}-\lambda _{L,j}}{\bar{\lambda }_{L,j}-\varepsilon _j\ }. \end{aligned}$$

The Temple lower bound expression can be directly obtained by inserting this identity into Eq. (16), giving

22 $$\begin{aligned} \varepsilon _j\ge \varepsilon ^{\text {Te}}_{L,J} \equiv \lambda _{L,j}-\frac{\sigma ^2_{L,j}}{\bar{\lambda }_{L,j}-\lambda _{L,j}}, \end{aligned}$$

where the previously unknown overlap $a_{L,jj}$ is replaced by the residual energy $\bar{\lambda }_{L,j}$. Although the residual energy is unknown as well, its lower bound estimates can be obtained directly. For example, from the definition in Eq.  (20) for $j=1$ it can be readily found that $\bar{\lambda }_{L,j}\ge \varepsilon _2$ for the ground state. A natural choice is then to substitute the Weinstein lower bound for the corresponding state; thus, a practical method is obtained for the calculation of lower bounds by Temple’s formula.

### SCLBT

By substituting $Q={\hat{Q}}_{L}$—the projection operator for the complementary space as defined in Eq. (5)—into the Cauchy–Schwartz inequality in Eq. (14) and using the residual energy, an improved lower bound inequality can be obtained:

23 $$\begin{aligned} \varepsilon _j\ge \lambda _{L,j}-\frac{\sigma ^2_{L,j}}{\left( \bar{\lambda }_{L,j}-\lambda _{L,j}\right) }\left[ 1+\frac{\sigma ^2_{L,j}}{\left( \lambda _{L,j}-\varepsilon _j\right) ^{2}}\sum _{\begin{array}{c} k\ne j \\ k=1 \end{array}}^{L}\frac{a_{L,jk}}{ a_{L,jj}}\right] ^{-1}. \end{aligned}$$

This bound is better than the Temple one, because the denominator in the second term is always greater than one. Although the overlaps $a_{L,jk}$ are unknown, they can be determined by considering Eq. (12) obtained from the Lánczos construct. Using the identity

24 $$\begin{aligned} \langle \varphi _{j}|{\hat{H}}|\Phi _{N,k}\rangle =\varepsilon _j\langle \varphi _{j}|\Phi _{N,k}\rangle =\lambda _{L,k}\langle \varphi _{j}|\Phi _{N,k}\rangle +\langle \varphi _{j}|{\hat{Q}}_{L}{\hat{H}}|\Phi _{N,k}\rangle , \end{aligned}$$

the overlaps can be rewritten in terms of eigenvalues, true energies, and variances as

25 $$\begin{aligned} \frac{a_{L,jk}}{a_{L,jj}}=\frac{\langle \varphi _{j}|\Phi _{N,k}\rangle ^{2}}{\langle \varphi _{j}|\Phi _{N,j}\rangle ^{2}} =\frac{\left( \lambda _{L,j}-\varepsilon _j\right) ^{2}\sigma ^2_{L,k}}{\left( \lambda _{L,k}-\varepsilon _j\right) ^{2}\sigma ^2_{L,j}}. \end{aligned}$$

Inserting this expression into Eq. (23) gives a lower bound as^50,51^

26 $$\begin{aligned} \varepsilon _j\ge & {} \lambda _{L,j}-\frac{\sigma ^2_{L,j}}{\left( \bar{\lambda }_{L,j}-\lambda _{L,j}\right) }\left[ 1+\sum _{k=1}^{j-1} \frac{\sigma ^2_{L,k}}{\left(\vphantom{\bar{\lambda }_{L,j}} \lambda _{L,j}-\lambda _{L,k}\right) ^{2}}+\sum _{k=j+1}^{L}\frac{ \sigma ^2_{L,k}}{\left(\vphantom{\bar{\lambda }_{L,j}} \lambda _{L,k}-\varepsilon _{L,j}\right) ^{2}}\right] ^{-1} \nonumber \\\equiv & {} \lambda _{L,j}-\frac{\sigma ^2_{L,j}}{\left( \bar{\lambda }_{L,j}-\lambda _{L,j}\right) \left(\vphantom{\bar{\lambda }_{L,j}} 1+T_{L,j} \right) }, \end{aligned}$$

where $T_{L,j}$ is defined as

27 $$\begin{aligned} T_{L,j}=\sum _{k=1}^{j-1} \frac{\sigma ^2_{L,k}}{\left( \lambda _{L,j}-\lambda _{L,k}\right) ^{2}}+\sum _{k=j+1}^{L}\frac{\sigma ^2_{L,k}}{\left( \lambda _{L,k}-\varepsilon _{L,j}\right) ^{2}} \end{aligned}$$

with $\varepsilon _{L,j}$ as the lower bound to the *j*th eigenvalue based on the *L*-dimensional space. This lower bound expression is a significant improvement over Temple’s lower bound. The lower bound provided by these expressions can be further improved by defining tighter bounds to the residual energy $\bar{\lambda }_{L,j}$ and to the overlap $a_{L,kk}$ as discussed below.

### *i*SCLBT

In order to improve the SCLBT, a better estimate to the residual energy $\bar{\lambda }_{L,j}$ needs to be found. Let us first define an energy $\eta _{L,L^*}$ with the condition

28 $$\begin{aligned} \varepsilon _{L^*}\le \eta _{L,L^*}\le \varepsilon _{L^*+1}, \end{aligned}$$

where $L^*$ is the highest energy level, for which Eq. (28) can be satisfied also for the associated Ritz eigenvalue $\lambda _{L,L^*}$. The value of $L^*$ is a parameter that can be varied according to the quality and validity of the Weinstein lower bound. Using these notations, the residual energy for the *j*th state can be rewritten as

29 $$\begin{aligned} \bar{\lambda }_{L,j}=\eta _{L,L^*}+\frac{\bar{\lambda }_{L,j}-\varepsilon _j}{\lambda _{L,j}-\varepsilon _j}\left[ \sum _{\begin{array}{c} k\ne j \\ k=1 \end{array}}^{L^*}a_{L,kj}\left( \varepsilon _k-\eta _{L,L^*}\right) +\sum _{k=L^*+1}^{\infty }a_{L,kj}\left( \varepsilon _k-\eta _{L,L^*}\right) \right] , \end{aligned}$$

which, considering that the second term with the infinite sum in Eq. (29) is positive, can be rewritten as

30 $$\begin{aligned} \bar{\lambda }_{L,j}\ge \eta _{L,L^*}-\frac{\bar{\lambda }_{L,j}-\varepsilon _j}{\lambda _{L,j}-\varepsilon _j}\sum _{\begin{array}{c} k\ne j \\ k=1 \end{array}}^{L^*}a_{L,kj}\left( \eta _{L,L^*}-\varepsilon _k\right) . \end{aligned}$$

The expression for the residual energy can be rearranged as

31 $$\begin{aligned} \bar{\lambda }_{L,j}-\lambda _{L,j}\ge \frac{\eta _{L,L^*}-\lambda _{L,j}-f_{L,j}}{\lambda _{L,j}-\varepsilon _j+f_{L,j}}\left( \lambda _{L,j}-\varepsilon _j\right) , \end{aligned}$$

where

32 $$\begin{aligned} f_{L,j}=\sum _{\begin{array}{c} k\ne j \\ k=1 \end{array}}^{L^*}\frac{a_{L,kj}}{a_{L,kk}}a_{L,kk}\left( \eta _{L,L^*}-\varepsilon _k\right) . \end{aligned}$$

For the working equations of the *i*SCLBT, estimates for $\eta _{L,L^*}$ and $a_{L,kk}$ need to be obtained. For the Lehmann expression, discussed in detail in the next section, it can be assumed that $L^*$ is the highest value for which the simple Weinstein lower bound condition given in Eq. (18) is satisfied, which implies that

33 $$\begin{aligned} \varepsilon _{L^*+1}\ge 2\lambda _{L,L^*}-\varepsilon _{L^*}\ge \lambda _{L,L^*}. \end{aligned}$$

However, for the *i*SCLBT $\eta _{L,L^*}=\lambda _{L,L^*}$ can be used instead of using the Weinstein lower bound for $\varepsilon _{L^*}$. Typically, there is a range $l_{min}\le L \le l_{max}$ of *L* values for which $L^*$ is the highest state for which the Weinstein lower bound is valid^6^. According to the Ritz theorem, $\lambda _{L,L^*}$ is a decreasing function of *L*. However, the highest possible value, $\lambda _{L,L^*}^{\mathrm {min}}$, is required, which still satisfies Eq. (18): this is the worst Ritz estimate for $\varepsilon _{L^*}$, that is still lower than $\varepsilon _{L^*+1}$. Thus, the validity condition for the *i*SCLBT for $L^*$ is weaker than the Weinstein lower bound condition, which enables to obtain lower bounds to excited states for lower *L* values compared to Lehmann’s theory and provides improved lower bound estimates.

For the SCLBT^50,51^, the upper bound for $a_{L,kk}$ is given as

34 $$\begin{aligned} a_{L,kk}\le \left( 1+\sum _{\begin{array}{c} j\ne k \\ j=1 \end{array}}^{L^*}\frac{a_{L,kj}}{a_{L,kk}}\right) ^{-1}, \end{aligned}$$

which, using the Lánczos construct, can be rewritten as

35 $$\begin{aligned} a_{L,kk}\le \frac{\sigma ^2_{L,k}}{\left( \lambda _{L,k}-\varepsilon _k\right) ^2}\left[ \frac{\sigma ^2_{L,k}}{\left( \lambda _{L,k}-\varepsilon _k\right) ^2}+T_{L,k}\right] ^{-1}. \end{aligned}$$

However, a tighter bound can be obtained by considering the Cauchy–Schwartz inequality (Eq. (14)) using the projection operator ${\hat{Q}}_L$ without introducing the residual energy such that

36 $$\begin{aligned} \left( \lambda _{L,k}-\varepsilon _k\right) ^2\le \frac{1-a_{L,kk}}{a_{L,kk}}\frac{\sigma ^2_{L,k}}{1+T_{L,k}}, \end{aligned}$$

where $T_{L,k}$ is given in Eq. (27). Rearranging this expression gives a new bound for $a_{L,kk}$ as

37 $$\begin{aligned} a_{L,kk}\le \frac{\sigma ^2_{L,k}}{\left( \lambda _{L,k}-\varepsilon _k\right) ^2}\left[ 1+\frac{\sigma ^2_{L,k}}{\left( \lambda _{L,k}-\varepsilon _k\right) ^2}+T_{L,k}\right] ^{-1}. \end{aligned}$$

The difference between this expression and Eq. (35) is the appearance of unity in the denominator, which provides a tighter upper bound to $a_{L,kk}$. When the Weinstein lower bound is valid, $\lambda _{L,k-1}\le \varepsilon _k\le \lambda _{L,k}$; thus, the maximal $a_{L,kk}$ as a function of $\varepsilon _k$ monotonically increases from 0 at $\varepsilon _k=\lambda _{L,k-1}$ to 1 at $\varepsilon _k=\lambda _{L,k}$. Using this expression and considering the above mentioned conditions for $\eta _{L,L^*}$, Eq. (32) can be maximized as

38 $$\begin{aligned} f_{L,j}^{\mathrm {max}}=\sum _{\begin{array}{c} k\ne j \\ k=1 \end{array}}^{L^*}\left( \lambda _{L,L^*}^{\mathrm {min}}-\varepsilon _{L,k}\right) \frac{\sigma ^2_{L,j}}{\left( \lambda _{L,j}-\varepsilon _{L,k}\right) ^2} \left[ 1+\frac{\sigma ^2_{L,k}}{\left( \lambda _{L,k}-\varepsilon _{L,k}\right) ^2}+T_{L,k}\right] ^{-1}, \end{aligned}$$

where the exact eigenvalues have been replaced by their lower bounds $\varepsilon _{L,k}$. The lower bound equation  (31) can be rewritten as

39 $$\begin{aligned} \varepsilon _j\ge \lambda _{L,j}-\frac{A_{L,j}^{\mathrm {max}}}{2}\left( 1+\sqrt{1+\frac{4f_{L,j}^{\mathrm {max}}}{A_{L,j}^{\mathrm {max}}}}\right) , \end{aligned}$$

where

40 $$\begin{aligned} A_{L,j}^{\mathrm {max}}=\frac{\sigma ^2_{L,j}}{\left( \lambda _{L,L^*}^{\mathrm {min}}-\lambda _{L,j}-f_{L,j}^{\mathrm {max}}\right) \left(\vphantom{\lambda _{L,L^*}^{\mathrm {min}}} 1+T_{L,j}\right) }. \end{aligned}$$

For the *i*SCLBT method, Eqs. (27) and (38)–(40) need to be solved simultaneously and iteratively. Using an initial estimate, such as the Weinstein bound, for the exact eigenvalues $\varepsilon _j$ in Eq. (27) the calculated lower bounds are substituted back into Eq. (27), until they converge. By increasing the highest considered state $L^*$, the lower bounds below this state can be improved.

### Lehmann theory

The Temple lower bound in Eq. (22) was obtained by using a specific choice of basis functions, which were the eigenfunctions of the Ritz eigenvalue. Lehmann constructed a method by which the basis can be optimized to obtain a linear combination of the states in the projected *L* dimensional space that maximizes the Temple lower bound. The Lehmann eigenvalue equation can be written as^22^

41 $$\begin{aligned} {\hat{P}}_{L}\left( {\hat{H}}-\rho {\hat{I}}\right) ^{2}|\Omega _{\kappa }\rangle =\kappa {\hat{P}}_{L}\left( {\hat{H}}-\rho {\hat{I}}\right) |\Omega _{\kappa }\rangle , \end{aligned}$$

where $\kappa$ is the Lehmann eigenvalue and $|\Omega _{\kappa }\rangle$ is the corresponding Lehmann eigenfunction in the projected *L* space. The parameter $\rho$ is known as the Lehmann pole and it is restricted by the condition

42 $$\begin{aligned} \varepsilon _{L^*+1}\ge \rho \ge \lambda _{L,L^*}\ge \varepsilon _{L^*}, \end{aligned}$$

where $L^{*}\le L$ is the highest state for which the inequality $\varepsilon _{L^*+1}\ge \lambda _{L,L^*}$ is satisfied. Thus, the Lehmann equation provides lower bounds only to states for which the corresponding Ritz eigenvalues are interleaving with the exact energies. Due to the square on the left-hand side of Eq. (41), this condition also implies that for lower bounds, the eigenvalue $\kappa$ is negative.

For an insightful analysis of the Lehmann equation, let us define a non-normalized state

43 $$\begin{aligned} |{\tilde{\Omega }}_{\kappa }\rangle =\left( {\hat{H}}-\rho {\hat{I}}\right) |\Omega _{\kappa }\rangle \end{aligned}$$

and rewrite the Lehmann equation as

44 $$\begin{aligned} \frac{1}{\kappa }=\frac{\langle {\tilde{\Omega }}_{\kappa }|\left( {\hat{H}}-\rho {\hat{I}}\right) ^{-1}|{\tilde{\Omega }}_{\kappa }\rangle }{\langle {\tilde{\Omega }} _{\kappa }|{\tilde{\Omega }}_{\kappa }\rangle }\ge \frac{1}{\varepsilon -\rho }, \end{aligned}$$

where the inequality follows by applying the Ritz theorem to the resolvent operator appearing on the left-hand side. Considering the restriction in Eq. (42) on the Lehmann pole, it can be written as

45 $$\begin{aligned} \varepsilon _{L^{*}}\ge \rho +\kappa _{L^{*}}\equiv \varepsilon _{\Omega _{L^{*}}}, \end{aligned}$$

where $\kappa _{L^{*}}$ is the largest of the negative Lehmann eigenvalues. Owing to the interleaving property of the Ritz eigenvalues, lower negative Lehmann eigenvalues provide lower bounds to all lower lying states. The connection with the Temple lower bound can be established by multiplying Eq. (41) by $\langle \Omega _{k}|$, which gives

46 $$\begin{aligned} \varepsilon _{\Omega _{\kappa }}=\langle \Omega _{\kappa }|{\hat{H}}|\Omega _{\kappa }\rangle -\frac{\sigma _{\Omega _{\kappa }}^{2}}{\left( \rho -\langle \Omega _{\kappa }|{\hat{H}}|\Omega _{\kappa }\rangle \right) }, \end{aligned}$$

with

47 $$\begin{aligned} \sigma _{\Omega _{\kappa }}^{2}=\langle \Omega _{\kappa }|{\hat{H}}^{2}|\Omega _{\kappa }\rangle -\langle \Omega _{\kappa }|{\hat{H}}|\Omega _{\kappa }\rangle ^{2}. \end{aligned}$$

Thus, according to the Ritz variational theorem, the Lehmann eigenfunction maximizes the Temple lower bound.

The Lehmann method requires the matrices of ${\hat{H}}^{2}$ and ${\hat{H}}$ in the *L*-space, and the lower bound eigenvalues can be obtained by the diagonalization of Eq. (41). According to the condition in Eq. (42), a non-trivial lower bound needs to be estimated for the state $\varepsilon _{L+1}$, which can typically be a Weinstein lower bound. Nevertheless, when a Lánczos basis is used, the full ${\hat{H}}^{2}$ matrix in the projected space is not required, but only the variances $\sigma ^2_{L,j}$ associated with the respective Ritz eigenvalues. This can be shown by multiplying Eq. (41) by $\langle \Phi _{L,k}|$, which gives

48 $$\begin{aligned} \langle \Phi _{L,k}|{\hat{H}}{\hat{Q}}_{L}{\hat{H}}|\Omega \rangle =\left( \lambda _{L,k}-\varepsilon _{\Omega }\right) \left( \rho -\lambda _{L,k}\right) \langle \Phi _{L,k}|\Omega \rangle , \end{aligned}$$

such that

49 $$\begin{aligned} \langle \Phi _{L,k}|\Psi _{L}\rangle H_{L,L+1}^{2}\langle \Psi _{L}|\Omega \rangle =\left( \lambda _{L,k}-\varepsilon _{\Omega }\right) \left( \rho -\lambda _{L,k}\right) \langle \Phi _{L,k}|\Omega \rangle . \end{aligned}$$

Multiplying Eq. (49) by $\langle \Psi _{L}|\Phi _{L,k}\rangle$ gives

50 $$\begin{aligned} \sigma ^2_{L,j}\langle \Psi _{L}|\Omega \rangle =\left( \lambda _{L,k}-\varepsilon _{\Omega }\right) \left( \rho -\lambda _{L,k}\right) \langle \Psi _{L}|\Phi _{L,k}\rangle \langle \Phi _{L,k}|\Omega \rangle . \end{aligned}$$

Rearranging and summing over all *k* from 1 to *L* gives an eigenvalue equation that is valid for the Lánczos construct as

51 $$\begin{aligned} \sum _{k=1}^{L}\frac{\sigma ^2_{L,j}}{ \left( \lambda _{L,k}-\varepsilon _{\Omega }\right) \left( \rho -\lambda _{L,k}\right) }=1. \end{aligned}$$

The variances can be obtained from Eq. (12), that is, all the information is in the matrix elements of the Lánczos representation of the Hamiltonian only.

## Results

For the comparison of the lower bounds calculated using the *i*SCLBT method with those of the SCLBT and Lehmann methods, the same two lattice systems were used as in Ref. 50. The first system was the Hubbard Hamiltonian^53^

52 $$\begin{aligned} H_{\mathrm {Hubbard}}=\epsilon \sum _{i,\sigma }c_{i,\sigma }c_{i,\sigma }^{\dagger }-t\sum _{\langle i, j\rangle }c_{i,\sigma }c_{j,\sigma }^{\dagger }+U\sum _i n_{i,\uparrow }n_{i,\downarrow }, \end{aligned}$$

where *i* denotes the lattice sites, $c_{i,\sigma }^{\dagger }$ and $c_{i,\sigma }$ are creation and annihilation operators for the electron in site *i* with spin $\sigma =\uparrow ,\downarrow$, respectively, $n_{i,\sigma }=c_{i,\sigma }^{\dagger }c_{i,\sigma }$ is the number operator for the spin state $\sigma$ on site *i*, $\epsilon$ is the “on-site” energy, which is set to zero in this case, *t* is the hopping energy between the nearest neighbors, and $U > 0$ is the Coulomb repulsion experienced by two electrons occupying the same site. The other model was the Heisenberg system^54^, describing a set of spin-1/2 particles on a lattice, given by the Hamiltonian

53 $$\begin{aligned} H_{\mathrm {Heisenberg}}=J\sum _{\langle i, j\rangle }{\mathbf {s}}_i{\mathbf {s}}_j, \end{aligned}$$

where ${\mathbf {s}}_i$ and ${\mathbf {s}}_j$ are the spin-1/2 operators on the lattice sites *i* and *j*, respectively, ${\langle i, j\rangle }$ denotes the nearest-neighbor pairs only, and *J* is the coupling (or “exchange”) constant weighting the “exchange term” ${\mathbf {s}}_i{\mathbf {s}}_j$. The detailed analysis of these systems is outside of the scope of this study: they were chosen because they are well suited for diagonalization using the Lánczos algorithm. The data used in this study was obtained using the ${\mathcal {H}}\Phi$ diagonalization software^55^ and available in Ref. 50.

**Figure 1:**
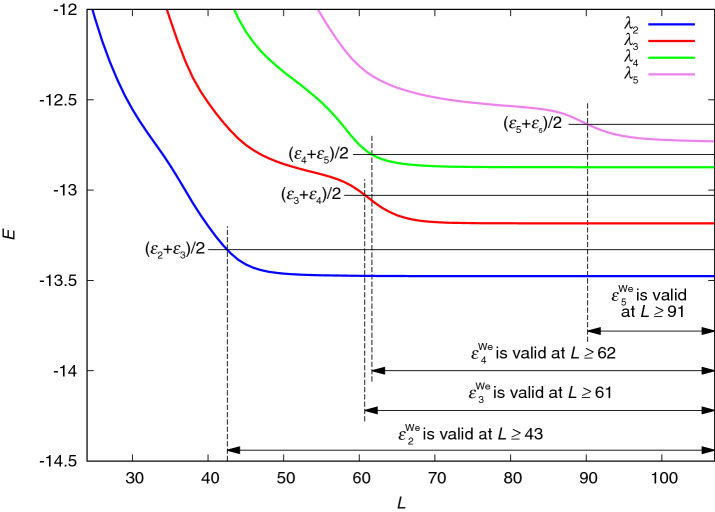
Ranges of validity of the Weinstein lower bound according to Eq. (18) for the Hubbard model. The blue, red, green, and violet lines indicate energy eigenvalues $\lambda _{2}$, $\lambda _{3}$, $\lambda _{4}$, and $\lambda _{5}$ as a function of dimensionality *L* of the Lánczos basis set, respectively. The graphs of the eigenvalue functions were intersected by the line of the condition of validity. As the true energy levels $\varepsilon _j$ are not known, the lowest eigenvalues $\lambda _{117,j}$ are used. The Weinstein lower bounds are valid from the *L* value greater than the point of intersection.

In these calculations, the lattices were always considered periodic and the simulation cell was limited to a finite number *N* of sites with periodic boundary conditions. The diagonal ($\alpha$) and off-diagonal ($\beta$) Lánczos coefficients of a Heisenberg square lattice with a unit cell of $5\times 6$ and of a Hubbard square lattice model at half-filling on a unit cell of $4 \times 4$ were used. A tridiagonal matrix was created from the $\alpha$ and $\beta$ coefficients and then, diagonalized using the double-precision Lapack *dsyev* subroutine^56^ for real, symmetric matrices. The $\lambda _{L,j}$ energy eigenvalues become unstable, that is, they started to increase and fluctuate, at $L > 118$ for the Hubbard and at $L>84$ for the Heisenberg model.

The Weinstein lower bounds were calculated using Eq. (17), and their ranges of validity^5^ were calculated according to Eq. (18). As the exact eigenvalues $\varepsilon _j$ were not known, the lowest stable eigenvalues $\lambda _{M,j}$ were used, at $M=117$ for the Hubbard and $M=83$ for the Heisenberg system. However, these values are sufficient, as only an estimation of the lowest *L* is required, from where the Weinstein lower bound can be considered valid. As $L^*=2$ is the lowest reference level for both the *i*SCLBT and Lehmann calculations, it is sufficient to determine the ranges of validity from $\lambda _{L,2}$. The graphs of the eigenvalues as a function of dimensionality *L* were intersected by the line from the validity condition in Eq. (18), than the next highest integer *L* provided the lowest limit of validity. Figures 1 and S1 show the ranges of validity of the Weinstein lower bounds for the Hubbard and Heisenberg models, respectively. These ranges were used to determine the range of validity of the SCLBT and Lehmann calculations. As can be seen in Figures 1 and S1 at $\lambda _{L,5}$ there are only a few remaining valid states; thus, the highest achievable level is $L^*=5$ for both the Hubbard and the Heisenberg systems.

Given the Weinstein lower bounds and so the corresponding residual energies, we first compared the performance of the *i*SCLBT described in the previous section with that of the previous SCLBT implementation given in Refs. 50,51. The quality of the lower bounds from these methods was compared using their gap ratios, defined as the ratio of the distances of the lower bound $\varepsilon _{L,j}$ and the eigenvalue $\lambda _{L,j}$ to the true energy level $\varepsilon _j$ as

54 $$\begin{aligned} \mathrm {Gap\;ratio}=\frac{|\varepsilon _{L,j}-\varepsilon _j|}{|\lambda _{L,j}-\varepsilon _j|}\simeq \frac{|\varepsilon _{L,j}-\lambda _{M,j}|}{|\lambda _{L,j}-\lambda _{M,j}|}, \end{aligned}$$

where $\lambda _{M,j}$ is the lowest stable eigenvalue.

For the ground-state residual energy, $\bar{\lambda }_{L,j}$, the first-excited state Weinstein lower bound $\varepsilon ^{\mathrm {We}}_2$ was used for the SCLBT method based on Eq.(26). In the *i*SCLBT method, the $\varepsilon _{L,j}$ bounds are calculated iteratively, by substituting the previously calculated lower bound back to the expression, until convergence is achieved. The residual energy $\bar{\lambda }_{L,j}$ was estimated from the Weinstein lower bound $\varepsilon ^{\mathrm {We}}_{L,j}$. The SCLBT^51^ improves Eq. (26) by self-consistently considering several states up to $L^*$; as shown in Ref. 52 increasing $L^*$ results in tighter lower bounds. For the *i*SCLBT method, Eqs. (27) and (38)–(40) were solved simultaneously and iteratively. The $\lambda _{L,L^*}^{\mathrm {min}}$ value in Eqs. (38) and (40) was determined based on the validity of the Weinstein lower bound: this was the highest eigenvalue where the Weinstein lower bound was still valid. In the first step of the iteration, the $\varepsilon _{L,j}$ values in Eq. (27) were estimated from the Weinstein lower bound $\varepsilon ^{\mathrm {We}}_{L,j}$ and the subsequent iteration steps use the previously calculated $\varepsilon _{L,j}$ values. Although any other estimated value could be used, such as the result $\varepsilon _{L-1,j}$ from the previous state, the calculation converged to the same value, regardless of the initial input; thus, the method does not explicitly rely on the Weinstein lower bound. The lowest $L_{\mathrm {ini}}$ value where proper convergence was achieved was slightly higher than the *L* value from where the Weinstein lower bound was valid.

**Figure 2:**
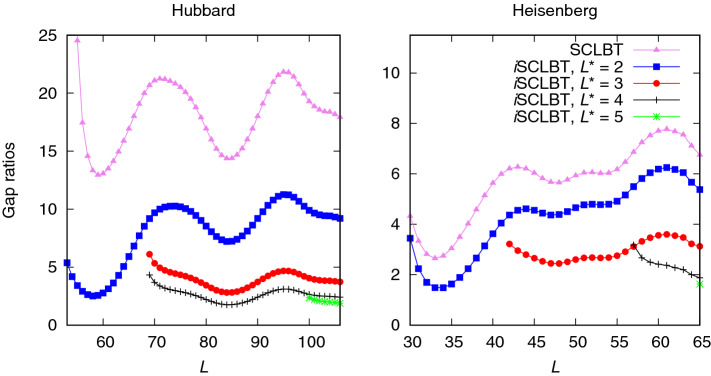
Comparison of ground-state lower bound gap ratios calculated by the SCLBT^50^ and by the improved version (*i*SCLBT) presented in this paper for the Hubbard (left panel) and Heisenberg (right panel) models. The violet line indicates the previous lower bound methodology and the blue, red, gray, and green lines indicate the *i*SCLBT calculations at $L^*=2$, $L^*=3$, $L^*=4$, and $L^*=5$, respectively, for both systems.

A comparison of the SCLBT and *i*SCLBT results at $L^*=2$, $L^*=3$, $L^*=4$, and $L^*=5$ is shown in Fig. 2. The noticeable variation of the functions is due to the fluctuation in the variances obtained from the Lánczos construct. As mentioned earlier, the SCLBT calculations start converging slightly later than the point where the Weinstein lower bound becomes valid. For example, the Weinstein lower bound for the third state $\varepsilon ^{\mathrm {We}}_3$ becomes valid at $L\ge 62$, as shown in Fig. 1, while the *i*SCLBT at $L^*=4$ starts converging at $L=69$, as shown in Fig. 2. When in Eq. (39) the ratio $4f_{L,j}^{\mathrm {max}}/A_{L,j}^{\mathrm {max}}<-1$, the lower bound becomes complex and thus, invalid. When the condition $4f_{L,j}^{\mathrm {max}}/A_{L,j}^{\mathrm {max}}>-1$ is satisfied, an appropriate convergence can be obtained for the lower bounds within a few iterations. At $L^*=2$ the *i*SCLBT simultaneously calculates lower bounds for the two lowest lying energy levels. It can be seen that the *i*SCLBT results are a significant improvement over the lower bounds from the SCLBT method^50^ even at $L^*=2$. With the increase in $L^*$, the *i*SCLBT results improve further and converge: the lower bound gaps for $L^*=4$ (gray line) and $L^*=5$ (green line) are very close to each other. The Heisenberg results for the ground-state lower bounds are significantly better than the Hubbard results, because the variance of the ground state Heisenberg eigenvalue is approximately three orders of magnitude smaller than that of the Hubbard system. While results from SCLBT^50^ are significantly better in the Heisenberg case than those of the Hubbard model case, the *i*SCLBT results are more accurate for both models, because the variances for the excited states are comparable.

**Figure 3:**
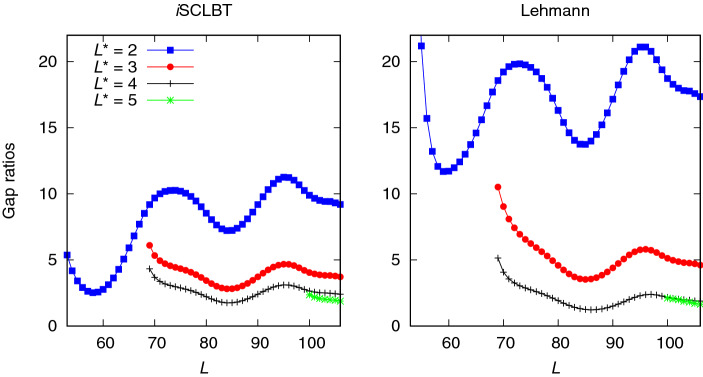
Comparison of ground-state lower bound gap ratios calculated by the *i*SCLBT and Lehmann methods for the Hubbard model. The blue, red, gray, and green lines indicate the *i*SCLBT and Lehmann calculations at $L^*=2$, $L^*=3$, $L^*=4$, and $L^*=5$, respectively.

The lower bound gap ratios calculated by the *i*SCLBT and the Lehmann theory were compared as well, using both lattice systems. Both methods can provide lower bounds not only for the ground state, but also for higher excited states. However, the number of treatable excited states is limited by the Lánczos construct, which provides reliable eigenvalues for the lowest lying energy levels only. While the *i*SCLBT does not depend on the Weinstein lower bound explicitly, the Lehmann theory relies on it. Theoretically, the Weinstein lower bound is a monotonically increasing function of *L* where it satisfies the condition of validity in Eq. (18). However, in practical calculations, due to the fluctuations of the variances with the increasing basis set size, the Weinstein lower bound function is not monotonic so that one uses the largest Weinstein lower bound. Specifically, if for basis set dimension $L+1$ the Weinstein lower bound is lower than for *L*, one uses the lower bound for *L*, etc., similar to the strategy employed in the SCLBT^50^. For the Lehmann theory, the corrected Weinstein lower bounds corresponding to $L^*$ were used for the Lehmann pole $\rho$ in Eq. (51). This equation provides $L^*$ roots, which were calculated using the Mathematica software (Wolfram Research). The *i*SCLBT and Lehmann results were considered in the same ranges. As can be seen in Fig. 1, $\lambda _{L,5}$ is the highest eigenvalue where the variance and the Weinstein lower bound calculated from it are valid. Thus, the highest achievable state is $L^*=5$, that is, the ground state and the four lowest lying excited states.

Figure 3 shows a comparison of the ground-state lower bound gap ratios for the Hubbard model calculated by the *i*SCLBT and Lehmann methods as a function of dimension *L* for different $L^*$ values, up to $L^*=5$. The fluctuation of the gap ratios for both methods is due to the fluctuation in the variances. As indicated by the blue line, the *i*SCLBT gap ratios at $L^*=2$ are significantly better than the corresponding Lehmann results. The Lehmann gap ratio at $L^*=2$ is slightly better than the results of the SCLBT indicated by the violet line shown in the left panel of Fig. 2 and the shape of the two graphs is similar. This is because both of these methods are based on the Weinstein lower bounds. As indicated by the red line in Fig. 3, the lower bounds improve significantly at $L^*=3$ for both the *i*SCLBT and Lehmann calculations; in this case, the *i*SCLBT results are still superior to those of the Lehmann method. However, while the *i*SCLBT can provide good quality lower bounds at lower *L* values, the Lehmann method requires a larger basis set size to achieve similar performance. The Lehmann results are comparable to those of the *i*SCLBT above $L\simeq 80$ in this case. At $L^*=4$ and $L^*=5$ both methods converge: the Lehmann lower bounds are slightly better than those calculated by the *i*SCLBT. The converged gap ratios at $L^*=5$ are 1.880 for the *i*SCLBT and 1.640 for the Lehmann method. The ground-state results indicate that the increase in $L^*$, the highest state to be considered, provides significant improvement in the lower bounds with a noticeable convergence for both lower bound methods, whose accuracy becomes similar.

**Figure 4:**
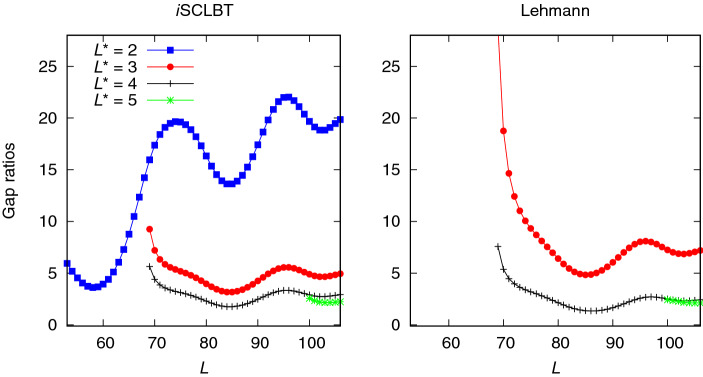
Comparison of first excited state lower bound gap ratios calculated by the *i*SCLBT and Lehmann methods for the Hubbard model. The blue line indicates *i*SCLBT calculation at $L^*=2$ and the red, gray, and green lines indicate results at $L^*=3$, $L^*=4$, and $L^*=5$, respectively, for both methods.

A comparison of the first excited state lower bound gap ratios for the Hubbard model obtained from the *i*SCLBT and the Lehmann theory is shown in Fig. 4. In the left panel, the blue line indicates lower bound gap ratios from the *i*SCLBT at $L^*=2$; however, while the *i*SCLBT method can calculate lower bounds for both states (ground and first-excited), the quality of the roots provided by the Lehmann theory for the first excited state at $L^*=2$ was extremely low and they monotonically increased with the increase in *L*. From $L^*=3$, the convergence of both methods is similar to that of the ground-state case, and the two methods exhibit similar performance for all $L^*$ values. At $L^*=3$ the *i*SCLBT results are significantly better than those of the Lehmann calculations, while at $L^*=4$ and $L^*=5$ the Lehmann method provides slightly tighter lower bounds. At $L^*>2$, the first excited state gap ratios are in the same range as those of the ground state and their converged gap ratios are similar as well: 2.236 for the *i*SCLBT and 2.113 for the Lehmann method.

**Figure 5:**
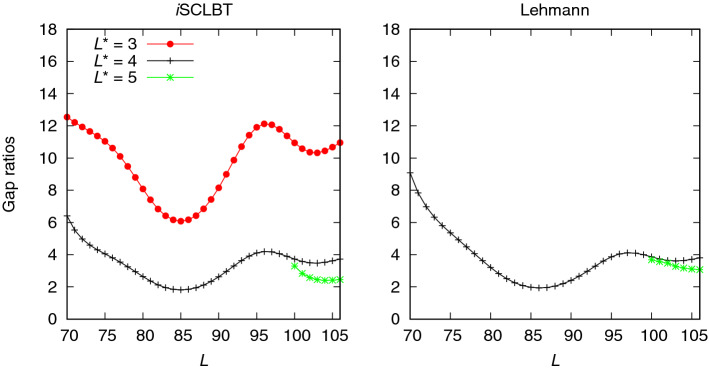
Comparison of second excited state lower bound gap ratios calculated by the *i*SCLBT and Lehmann methods for the Hubbard model.The red line indicates *i*SCLBT calculation at $L^*=3$ and the gray and green lines indicate results at $L^*=4$ and $L^*=5$, respectively, for both methods.

**Figure 6:**
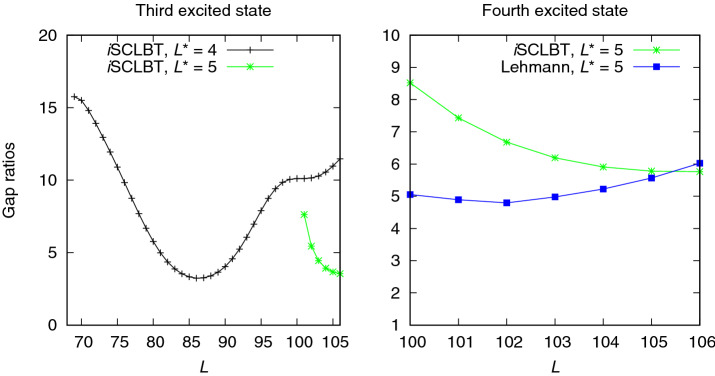
Results for the Hubbard model for the third and fourth excited states. Left panel: comparison of third excited state lower bound gap ratios calculated by the *i*SCLBT method. The gray and green lines indicate results at $L^*=4$ and $L^*=5$, respectively. In this case the Lehmann results were of very low quality and thus, they are not included in the figure. Right panel: fourth excited state lower bound gap ratios for the *i*SCLBT (green line) and Lehmann (blue line) methods at $L^*=5$.

The second excited state results shown in Fig. 5 exhibit similar behavior: the quality of the roots from the Lehmann results was very low at $L^*=3$. In this case, the *i*SCLBT results are slightly better than the Lehmann bounds. At $L^*>4$ the gap ratios are in the range of those of the ground and first excited states. The converged gap ratios are 2.454 for the *i*SCLBT and 3.071 for the Lehmann calculations. Figure 6 shows results for the third and fourth excited states for the Hubbard model. The quality of the Lehmann results was very low for the third excited state (left panel) even at $L^*=4$. The quality of the corresponding Weinstein lower bound directly determines the quality of the Lehmann bound, as it explicitly depends on it. In this case the Weinstein bound of the fourth excited state, $\varepsilon ^{\mathrm {We}}_{L,4}$ has a local minimum at $L=90$ and even when corrected as discussed above, it provides poor quality results. However, the *i*SCLBT can still provide good quality lower bounds regardless of the quality of the Weinstein lower bound, as shown in the left panel of Fig. 6. The convergence behavior of the *i*SCLBT result is similar to that of the previous cases, with a final gap ratio of 3.545. As can be seen in Fig. 1, the highest energy level for which lower bounds could be obtained for the Hubbard model was the fourth excited state. The right panel of Fig. 6 shows lower bound gap ratios for both methods at $L^*=5$. Although the Weinstein lower bound $\varepsilon ^{\mathrm {We}}_{L,5}$ has a local minimum as well, for the final few states it results in good quality Lehmann bounds. The converged, final gap ratios are 5.764 for the *i*SCLBT and 6.024 for the Lehmann method.

As can be seen in Figs. 3, 4, 5 and 6, the increase in $L^*$ always results in an improvement of the lower bound for both methods. While the *i*SCLBT can provide lower bounds for all energy levels at a given $L^*$ reference level, the quality of the lower bounds calculated by the Lehmann method at the lowest $L^*$ value is extremely low; furthermore, the lower bounds are sharply increasing with the increase in dimensionality *L*. Generally, the Lehmann method can only provide $L^*-1$ appropriate roots for a calculation using $L^*$ states. The convergence behavior of the *i*SCLBT and Lehmann methods is similar in all cases. The Ritz gaps together with the final *i*SCLBT and Lehmann gap ratios for the Hubbard model are summarized in Table 1. The final values were calculated at $L=106$, where the lower bound gaps were still numerically reliable. It can be seen that as one goes up the ladder of states all gaps and gap ratios increase. As mentioned before, the quality of the third excited state gap ratio calculated by the Lehmann method is very low.

The same calculations were performed for the Heisenberg model; the results can be found in the Supplementary Information. As shown in Fig. S1, the Lánczos construct provides less eigenvalues for the Heisenberg model; thus, the number of treatable energy levels is lower. The Heisenberg results further confirm the convergence behavior demonstrated in the Hubbard system. The ground-state results shown in Fig. S2 are better than those of the Hubbard model because, as discussed before, the Heisenberg eigenvalues have smaller variances for the ground state. Similar to the Hubbard case, the *i*SCLBT is noticeably better at $L^*=2$ and $L^*=3$. The converged gap values are 1.625 for the *i*SCLBT and 1.250 for the Lehmann method. For the first excited state lower bound shown in Fig. S3, the *i*SCLBT at $L^*=2$ provides better gap ratios at low *L* values. This does not indicate that the lower bounds are better in this region; it rather shows that the Ritz gaps are still wide at low *L* values. From $L=70$ the two methods exhibit similar convergence characteristics as that of the ground-state case: at $L^*=3$ the *i*SCLBT is better than the Lehmann result, while at higher $L^*$ values the Lehmann method is slightly better. The final lower bound gap values are 2.148 for the *i*SCLBT and 1.716 for the Lehmann method. The second and third excited state lower bounds can be seen in Figs. S4 and S5, respectively. The behavior and convergence of the *i*SCLBT and Lehmann results for the second excited state are similar to those of the Hubbard model. The converged lower bound gap ratios are 2.363 and 1.991 for the *i*SCLBT and Lehmann methods, respectively. In the Heisenberg model, the Lehmann method can provide reasonable lower bounds for the third excited state; however, these values are monotonically increasing with dimensionality *L*. As there is only one remaining value at $L^*=5$, these results cannot be considered fully converged. The Ritz and lower bound gaps and final gap ratios for both methods are summarized in Table 2. It should be noted that for the fourth excited state only one point could be used, as the Lánczos construct provided only a few numerically reliable eigenvalues.

**Table 1:** Ritz gaps and the final *i*SCLBT and Lehmann gap ratios at $L^*=5$ calculated at $L=106$ for the Hubbard model.

State	Ground	1st excited	2nd excited	3rd excited	4th excited
Ritz gap	$4.441\times 10^{-14}$	$2.414\times 10^{-12}$	$1.663\times 10^{-8}$	$4.072\times 10^{-6}$	$3.970\times 10^{-3}$
Final *i*SCLBT gap ratio	1.880	2.236	2.454	3.545	5.764
Final Lehmann gap ratio	1.640	2.113	3.071	18.516	6.024

**Table 2:** Ritz gaps and the final *i*SCLBT and Lehmann gap ratios at $L^*=5$ calculated at $L=65$ for the Heisenberg model.

State	Ground	1st excited	2nd excited	3rd excited	4th excited
Ritz gap	$2.842\times 10^{-14}$	$4.058\times 10^{-10}$	$6.225\times 10^{-8}$	$1.338\times 10^{-3}$	$1.165\times 10^{-2}$
Final *i*SCLBT gap ratio	1.625	2.148	2.363	6.687	8.796
Final Lehmann gap ratio	1.250	1.716	1.991	7.332	3.549

## Discussion

In this paper, a further improvement of the existing SCLBT method was presented, termed *i*SCLBT. The lower bounds for the Ritz eigenvalues obtained from the *i*SCLBT were compared with those from the SCLBT and from the Lehmann methods. The framework provided by the Lánczos construct enabled the comparison of these methods on an equal formal footing. For the comparison, two model systems, a 5 $\times$ 6 Heisenberg and a 4 $\times$ 4 Hubbard lattice Hamiltonian were used. The eigenvalues and variances were determined by the Lánczos algorithm by using the ${\mathcal {H}}\Phi$ diagonalization software. By defining tighter bounds for the residual energy and for the diagonal elements of the overlap, the *i*SCLBT was improved over its previous implementation. First, the *i*SCLBT was compared with the SCLBT method for the ground-state energy. The improved theory provided significantly better lower bounds even at $L^*=2$. Then, the lower bounds for the low-lying energy levels were calculated using the *i*SCLBT and the Lehmann method up to the fourth excited state. The effect of the highest considered level $L^*$ on the quality of the lower bounds was analyzed. Based on the analysis of the results, the following conclusions can be drawn:

- The definition of tighter bounds for the residual energy and for the diagonal elements of the overlap further improved the SCLBT.
- Both the *i*SCLBT and the Lehmann method can provide lower bounds that are significantly better than the Weinstein or Temple bounds.
- The quality of lower bounds improves with the increase in $L^*$, the highest considered level.
- The *i*SCLBT and Lehmann methods exhibit similar performance and convergence behavior. This is not unexpected, considering that in Ref. 42, using a finite Hamiltonian construct and assuming a Lánczos basis set, we could find conditions under which the two theories would be identical. However, in general, and under the conditions of the present theory which differs from the one presented in Ref. 42, the Lehmann theory and *i*SCLBT are formally different.
- Compared to the *i*SCLBT, the Lehmann method requires a larger basis set and, as the Lehmann pole is estimated from the Weinstein lower bound, the quality of the Lehmann bounds is strongly affected by the quality of the Weinstein lower bound.
- The numerical implementation of *i*SCLBT is simpler than that of the Lehmann method and does not require the Weinstein lower bounds.

Both the *i*SCLBT and Lehmann methods are suitable to provide high quality lower bounds for the low-lying energy levels for the studied lattice systems. The number of treatable Ritz eigenvalues is determined by the size of the Lánczos basis set.

## Supplementary Information

Supplementary Information.

## Data Availability

Data and numerical codes are available from the authors upon reasonable request.
