# Supplementary material for: Comparison of an improved self-consistent lower bound theory with Lehmann’s method for low-lying eigenvalues
Source: Sci Rep. 2021 Dec 6;11:23450. doi: 10.1038/s41598-021-02473-y (PMC8648772; doi:10.1038/s41598-021-02473-y)
Supplement: Supplementary file 1 — Supplementary Information. [file 41598_2021_2473_MOESM1_ESM.pdf]

Supplementary Information

The Supplementary Information contains figures showing results obtained using the Heisenberg model.

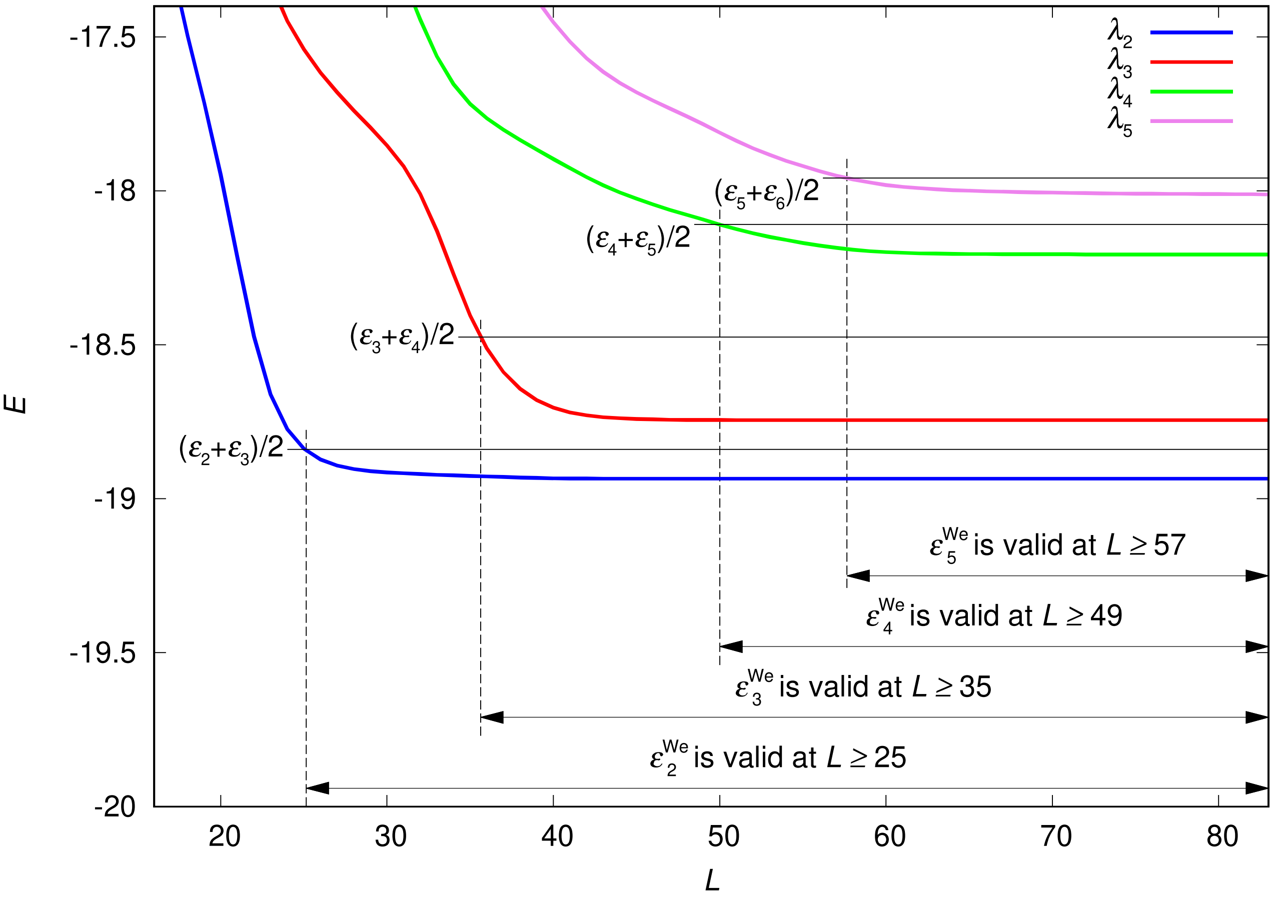

Figure S1. [redacted] basis set used. The graphs of the eigenvalue functions were intersected by the line of the condition of validity. As the true energy levels ε_j are not known, the lowest eigenvalues $\lambda_{83,j}$ are used. The Weinstein lower bounds are valid from the L value greater than the point of intersection.

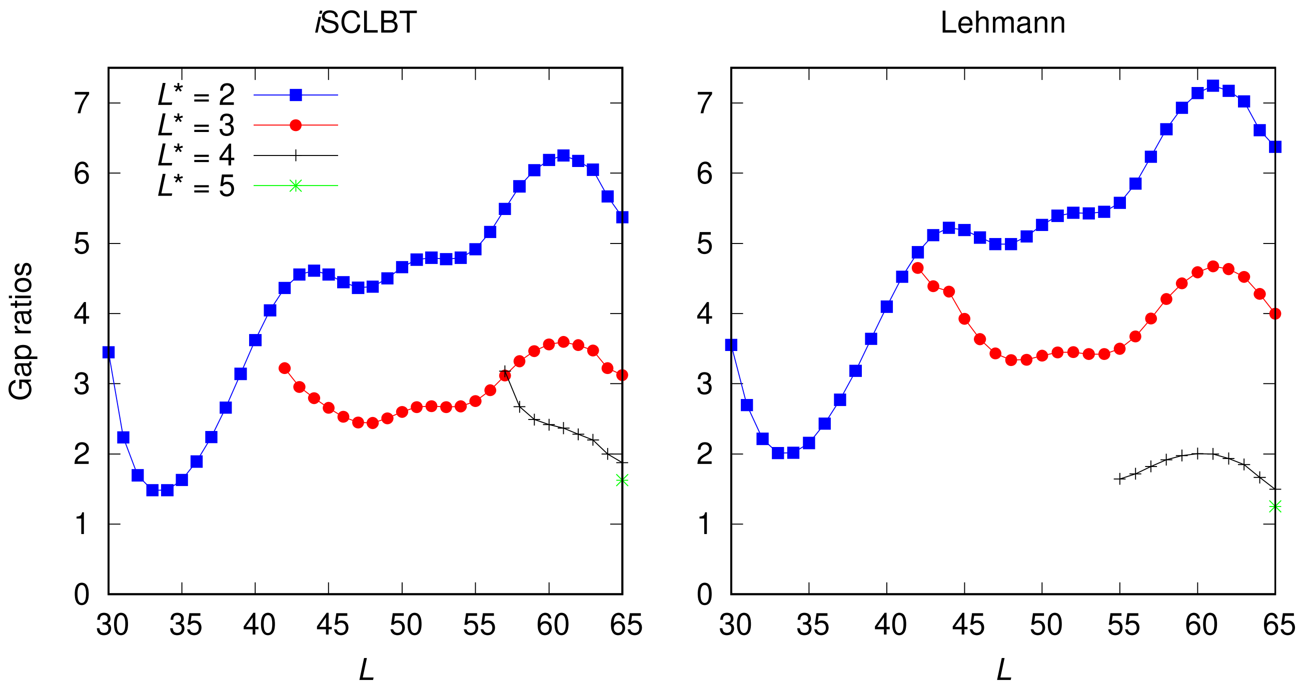

Figure S2. Comparison of ground-state lower bound gap ratios calculated by the iSCLBT and Lehmann methods for the Heisenberg model. The blue, red, gray, and green lines indicate the iSCLBT and Lehmann calculations at $L^* = 2$, $L^* = 3$, $L^* = 4$, and $L^* = 5$, respectively.

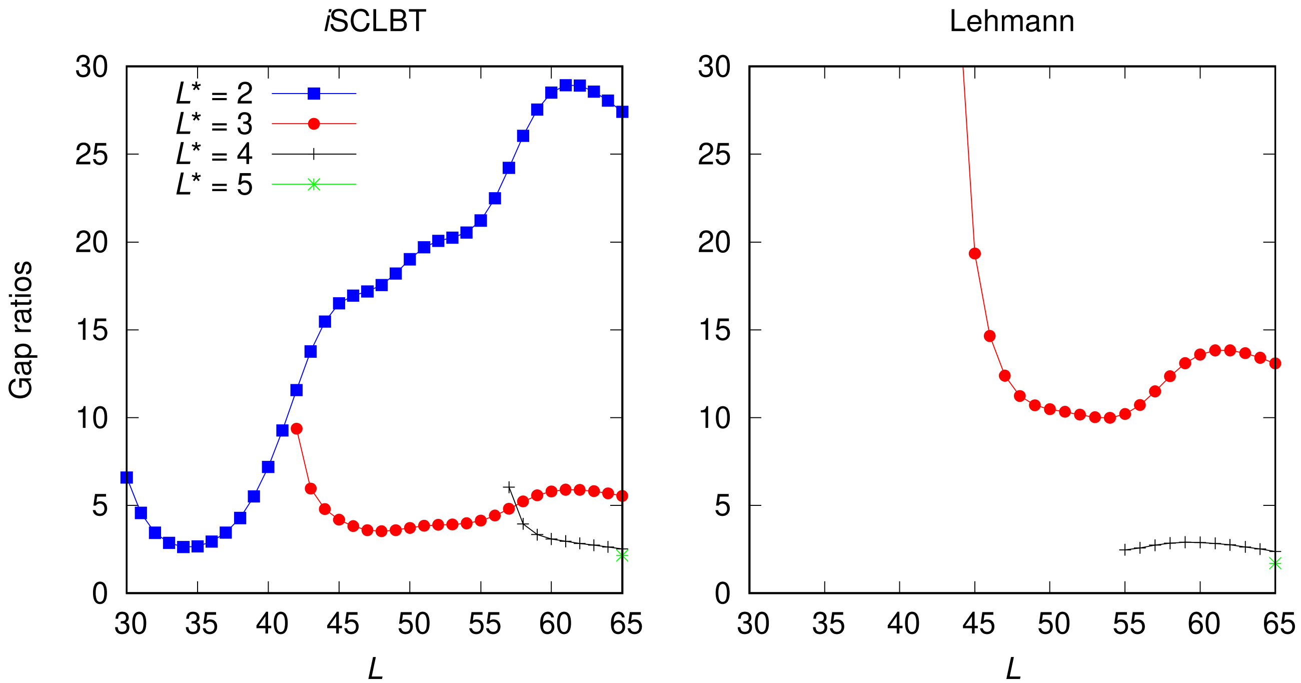

Figure S3. Comparison of first excited state lower bound gap ratios calculated by the [redacted] Heisenberg model. The blue line indicates iSCLBT calculation at $L^* = 2$ and the red, gray, and green lines indicate results at $L^* = 3$, $L^* = 4$, and $L^* = 5$, respectively, for both methods.

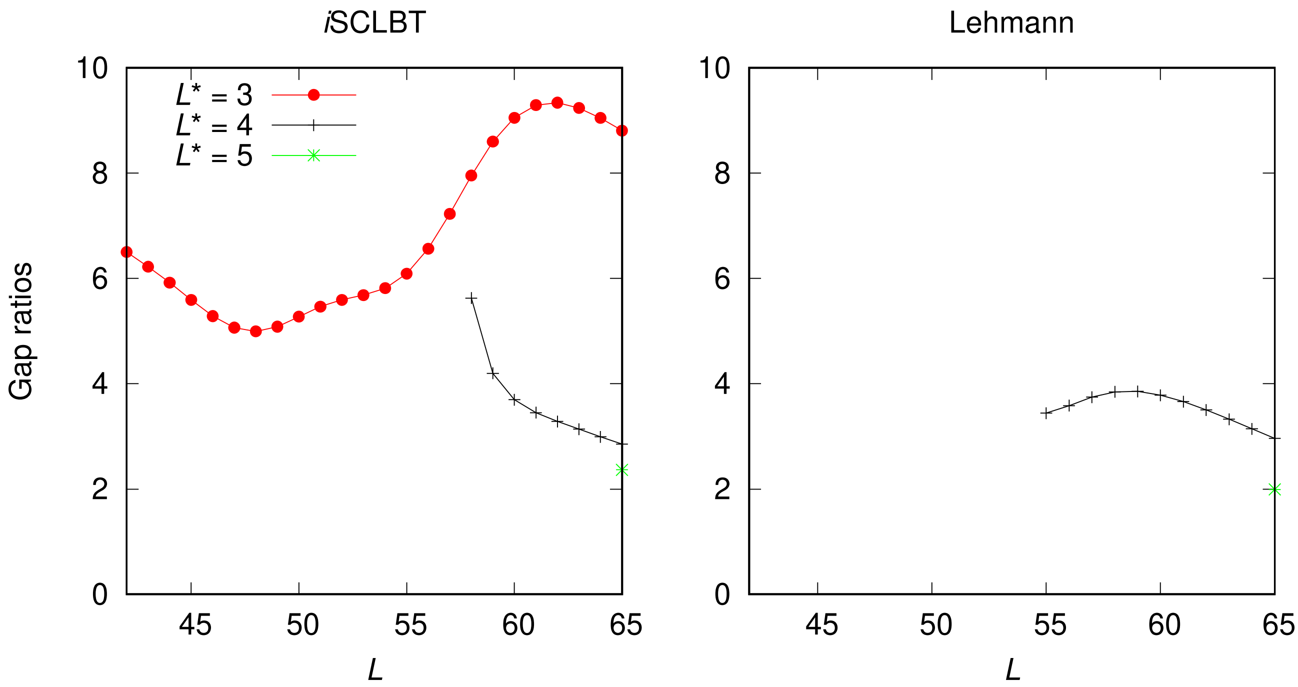

Figure S4. [redacted] the Heisenberg model. The red line indicates *i*SCLBT calculations at $L^* = 3$ and the gray and green lines indicate results at $L^* = 4$ and $L^* = 5$, respectively, for both methods.

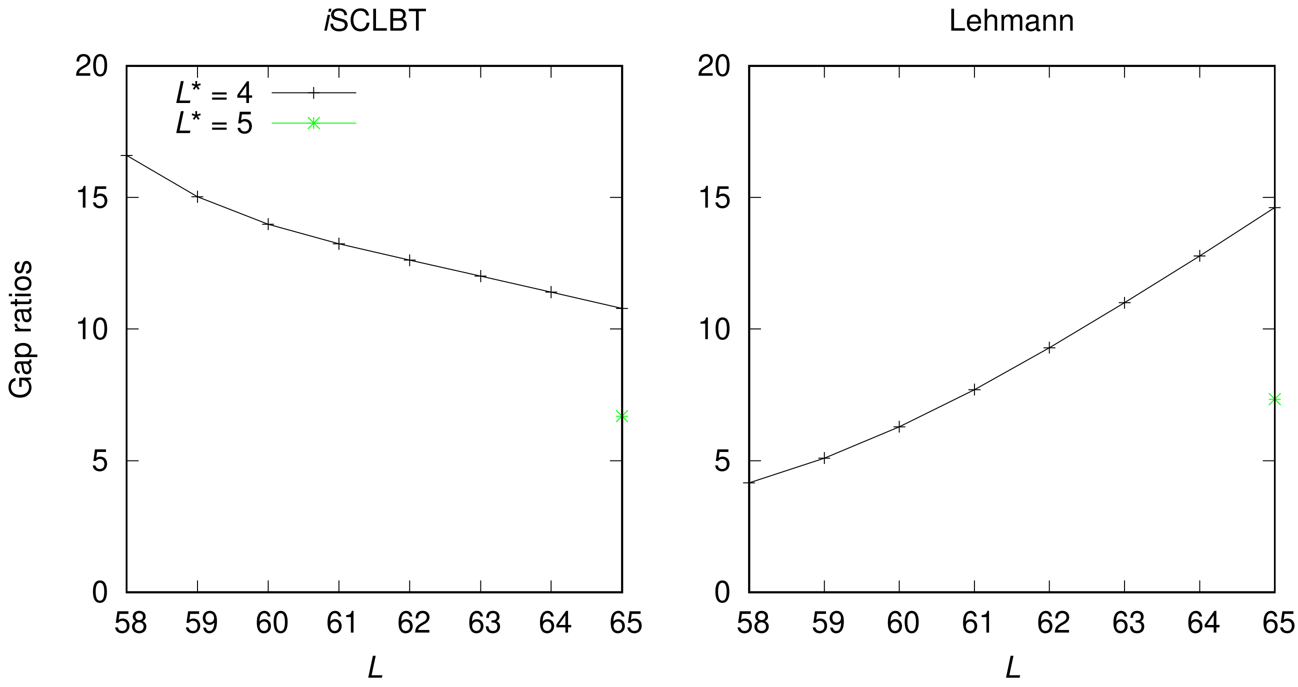

Figure S5. [redacted] Heisenberg model. The gray and green lines indicate results at $L^* = 4$ and $L^* = 5$, respectively, for both methods.
